# Identification of key genes in invasive clinically non-functioning pituitary adenoma by integrating analysis of DNA methylation and mRNA expression profiles

**DOI:** 10.1186/s12967-019-02148-3

**Published:** 2019-12-03

**Authors:** Sen Cheng, Weiyan Xie, Yazhou Miao, Jing Guo, Jichao Wang, Chuzhong Li, Yazhuo Zhang

**Affiliations:** 1 Beijing Neurosurgical Institute, Capital Medical University, Beijing, 100070 China; 2 Beijing Neurosurgical Institute, Beijing Tiantan Hospital Affiliated to Capital Medical University, Beijing Institute for Brain Disorders Brain Tumor Center, China National Clinical Research Center for Neurological Diseases, Key Laboratory of Central Nervous System Injury Research, Beijing, 100070 China; 3 People’s Hospital of Xin Jiang Uygur Autonomous Region, Urumqi, 830001 China

**Keywords:** Clinically non-functioning pituitary adenoma, DNA methylation, Invasion

## Abstract

**Background:**

Tumor surrounding the internal carotid artery or invading to the cavernous sinus is an important characteristic of invasive pituitary adenoma, and a pivotal factor of tumor residue and regrowth. Without specific changes in serum hormone related to the adenohypophyseal cell of origin, clinically non-functioning pituitary adenoma is more likely to be diagnosed at invasive stages compared with functioning pituitary adenoma. The underlying mechanism of tumor invasion remains unknown. In this study, we aimed to identify key genes in tumor invasion by integrating analyses of DNA methylation and gene expression profiles.

**Method:**

Genome-wide DNA methylation and mRNA microarray analysis were performed for tumor samples from 68 patients at the Beijing Tiantan Hospital. Differentially expressed genes and methylated probes were identified based on an invasive vs non-invasive grouping. Differentially methylated probes in the promoter region of targeted genes were assessed. Pearson correlation analysis was used to identify genes with a strong association between DNA methylation status and expression levels. Pyrosequencing and RT-PCR were used to validate the methylation status and expression levels of candidate genes, respectively.

**Results:**

A total of 8842 differentially methylated probes, located on 4582 genes, and 661 differentially expressed genes were identified. Both promoter methylation and expression alterations were observed for 115 genes with 58 genes showing a negative correlation between DNA methylation status and expression level. Nineteen genes that exhibited notably negative correlations between DNA methylation and gene expression levels, are involved in various gene ontologies and pathways, or played an important role in different diseases, were regarded as candidate genes. We found an increased methylation with a decreased expression of PHYHD1, LTBR, C22orf42, PRR5, ANKDD1A, RAB13, CAMKV, KIFC3, WNT4 and STAT6, and a decreased methylation with an increased expression of MYBPHL. The methylation status and expression levels of these genes were validated by pyrosequencing and RT-PCR.

**Conclusions:**

The DNA methylation and expression levels of PHYHD1, LTBR, MYBPHL, C22orf42, PRR5, ANKDD1A, RAB13, CAMKV, KIFC3, WNT4 and STAT6 are associated with tumor invasion, and these genes may become the potential genes for targeted therapy.

## Background

Pituitary adenomas (PA) are benign neuroendocrine tumors arising from adenohypophyseal cells and account for 10–15% of all primary intracranial neoplasms [1, 2]. Compared with functioning pituitary adenoma (FPA), clinically non-functioning pituitary adenoma (NFPA) often shows no specific serum hormone changes [3]. When the mass effect appears, it is possible that the tumor may have surrounded the internal carotid artery and invaded the cavernous sinus, which is the main cause of residual tumor and postoperative regrowth [4].

The overall rate of tumor invasion into the cavernous sinus is 35%, and the understanding of the mechanisms underlying the pituitary adenoma invasiveness is still far from comprehensive [5]. Nevertheless, studies have indicated that the pituitary tumorigenesis and tumor invasiveness may not be driven by gene mutations [6, 7] but rather by other regulatory mechanisms. Abnormalities of DNA methylation status of cytosine phosphate guanosine (CpG) islands in gene promoter regions are recognized to play a key role in regulating transcription and are closely associated with diverse diseases [8–11]. For example, some studies have reported methylation status alterations in NFPA tumorigenesis, but failed to uncover the changes in methylation involved in tumor invasiveness [12, 13]. Although these studies have identified the methylation changes in NFPA, limited work have been performed to connect these changes with whole-transcriptome changes. Furthermore, the blueprint of epigenetics cooperating with the transcriptome in NFPA has not been fully evaluated.

In this study, integrated analyses of paired whole-genome DNA methylation and gene expression microarray were performed. We found eleven genes that play an important role in the invasive behavior of NFPA. Pyrosequencing and RT-PCR were then utilized to validate the DNA methylation status and gene expression level, respectively. We aimed to identify gene expression epigenetically regulated by methylation changes in invasive NFPA, and hope to obtain a better understanding of the invasive behavior of NFPA.

## Materials and methods

### Patients and samples

This study retrospectively enrolled 68 patients aged 25–74 years who were diagnosed with NFPA from October 2007 to July 2016. All patients underwent enhanced head MRI scan before and after trans-sphenoidal surgery in order to assess the maximum tumor diameter, anatomical location and tumor resection grade. Tumor invasion was defined by tumor extension beyond the lateral tangent of the intra- and supra-cavernous internal carotid artery (Grade 3 and 4) as defined by Knosp et al. [14]. Only patients who were clinically diagnosed with and pathologically confirmed as having gonadotroph adenoma and null-cell adenoma were included in the study. All tumor samples were collected from the Neurosurgery Department of the Beijing Tiantan Hospital. Tumor samples were immediately placed into a sample tube, frozen in liquid nitrogen and stored. A total of 68 pituitary adenoma samples, including 46 invasive and 22 non-invasive tumors, were used for following whole-genome DNA methylation and mRNA microarray analysis.

### Whole-genome DNA methylation microarray

DNA was extracted and purified with DNeasy Blood & Tissue Kit (Qiagen, Germany). The bisulfite conversion of the DNA was performed using EZ DNA Methylation-Gold™ Kit (Zymo, USA). The total DNA methylation status of 68 NFPA samples was assayed by Illumina Infinium MethylationEPIC 850K BeadChip. The DNA methylation microarray experiment was performed at Shanghai Biotechnology Corporation following the manufacturer’s instructions of Illumina. Probes located on the sex chromosomes, bound to multiple genomic locations or associated with a known SNP were excluded [15]. Probes that failed to be detected above background were also removed from the research. The bio-conductor R package *minfi* was used to control quality and normalize the raw data [16]. The methylation status of different CpG sites was calculated with the average-difference β-value (Δβ), where beta-value (β) is between 0 (unmethylated) and 1 (completely methylated). Differentially methylated probes (DMPs) that showed a |∆β| of 0.1 and an adjusted p-value < 0.05 were regarded as significantly differentially methylated. Then a separate average DNA methylation β-value of the DMPs in the gene promoter and non-promoter region was employed to represent the methylation status, respectively.

### Whole-genome mRNA microarray

RNA was isolated and purified with the mirVana™ miRNA Isolation Kit (Ambion, USA), then amplified and labeled with Low Input Quick Amp WT Labeling Kit (Agilent Technologies, USA), following the manufacturer’s instructions. Then, purified RNA was used to generate fluorescence-labeled cRNA targets for SBC human ceRNA array V1.0 (4 × 180 K). The labeled cRNA targets were then hybridized and scanned with an Agilent Microarray Scanner (Agilent Technologies, USA). The data were extracted with Feature Extraction software 10.7 (Agilent Technologies, USA), and the R package *limma* was used to normalize the raw data [17]. The microarray experiments were performed at Shanghai Biotechnology Corporation following the protocol of Agilent Technologies. Differential gene expression was analyzed with a t-test in *limma* of the R package [18]. The fold change method was applied to estimate the differential significance of mRNAs. In our study, invasion-related differentially expressed genes (DEGs) in 68 NFPA samples were defined as the genes with a fold change > 1.5 or < 0.67, a p-value < 0.05 and a FDR value < 0.25.

### Integrated analysis

A two-step analysis process was performed to integrate promoter DNA methylation in the promoter region with gene expression. First, we located all DMPs (unfiltered) for targeted genes to identify differentially methylated genes (DMGs) between invasive and non-invasive tumors. Second, for the both differentially methylated and differentially expressed genes, we used Pearson correlation analysis to examine whether there was a strong association between the DNA methylation status and expression levels. In this study, we applied Pearson correlation coefficient (PCC) and p-values to investigate the correlation and significance between mRNA expression and DNA methylation with the function cor.test in R. A significantly negative correlation was considered if PCC < − 0.2 and p-value < 0.05.

To infer potential biological processes and pathways of invasion-related genes, the DAVID Bioinformatics Tool (v6.8) was employed to perform functional enrichment analysis using the Gene Ontology (GO) and Kyoto Encyclopedia of Genes and Genomes (KEGG). Biological processes and pathways were considered significant at p-value < 0.05.

### Pyrosequencing assay

Pyrosequencing assay was used to access the level of DNA methylation of the promoter regions of target genes. Genomic DNA from NFPA samples was extracted with QIAamp DNA Mini Kit (Qiagen, Germany). For each sample, 0.5 μg DNA was used to for bisulfite conversion with the EpiTect Bisulfite kit (Qiagen, Germany). 1 μl of 10 μl of eluted bisulfite converted DNA was used for performing PCR with PyroMark PCR Kit (Qiagen, Germany), according to the manufacturer’s instructions. The methylation status of each gene was assessed as the percentage of average methylation at targeted CpG sites. The PCR primer designed for pyrosequencing is shown in Additional file 1: Table S8.

### Real‑time quantitative reverse transcription polymerase chain reaction (RT-PCR)

Extraction and purification of total RNA were carried out as described above. RT-PCR analyses were performed with PrimerScript RT reagent Kit (Takara, China). Quantitative real-time PCR was performed using LightCycler 480 SYBR Green I Master (Roche, Switzerland) with the ABI 9700 PCR system (Applied Biosystems, USA). Reactions were run at 95 °C for 10 min followed by 40 cycles of 95 °C for 10 s and 60 °C for 30 s. Gene expression was measured by the standard curve method and normalized to the level of β-actin; the 2^−ΔΔct^ method were used for calculations [19]. The PCR primer sequences are shown in Additional file 1: Table S9.

### Statistical analysis

Statistical analyses were performed in R (version 3.1) and IBM SPSS Statistics for Windows (v 21.0). Student’s t test was applied for comparison of two groups and for pyrosequencing and RT-PCR assay, respectively. All data are presented as the mean ± standard deviation (SD). Differences were considered to be statistically significant if p < 0.05.

## Results

### Whole-genome DNA methylation analysis

**Fig. 1:**
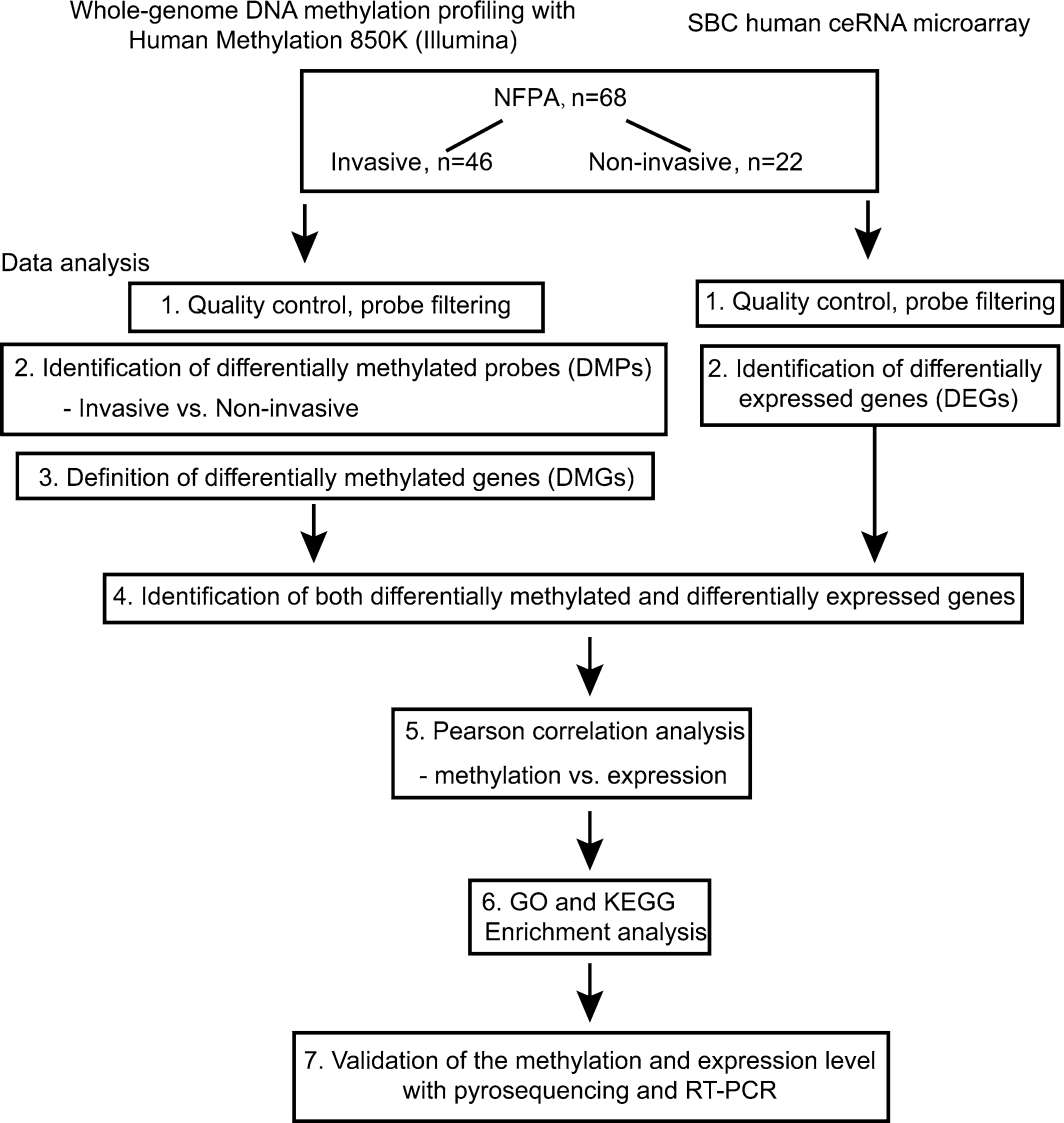
Flowchart of this study

The flowchart of the study is shown in Fig. 1. Patients were separated into invasive and non-invasive groups based on the criteria mentioned above. The clinical characteristics of these 68 patients are shown in Table 1 and detailed information of each patient is provided in Additional file 1: Table S1. We compared the DNA methylation patterns in those two groups by whole-genome DNA methylation microarray (invasive vs non-invasive tumor). After filtering the raw data (602,698 probes remained) and performing statistical analysis, 8842 probes that showed significant changes with |∆β| > 0.1 between invasive and non-invasive groups were included for downstream analyses.

**Table 1:** Clinical characteristics of 68 patients with NFPA

	N	Percentage (%)
Gender		
Male	35	51.47
Female	33	48.53
Age		
Mean	50.28 ± 11.47	
Median	51.5	
Tumor volume		
Macro-	48	70.59
Giant	20	29.41
Invasive		
Yes	46	67.65
No	22	32.35
Subtype		
Null cell	45	66.18
Gonadotroph	23	33.82
Resection		
GTR	33	48.53
NGTR	35	51.47

*Macro* macro-adenoma (1–4 cm), *Giant* giant adenoma (> 4 cm), *GTR* gross total resection, *NGTR* non-gross total resection

**Fig. 2:**
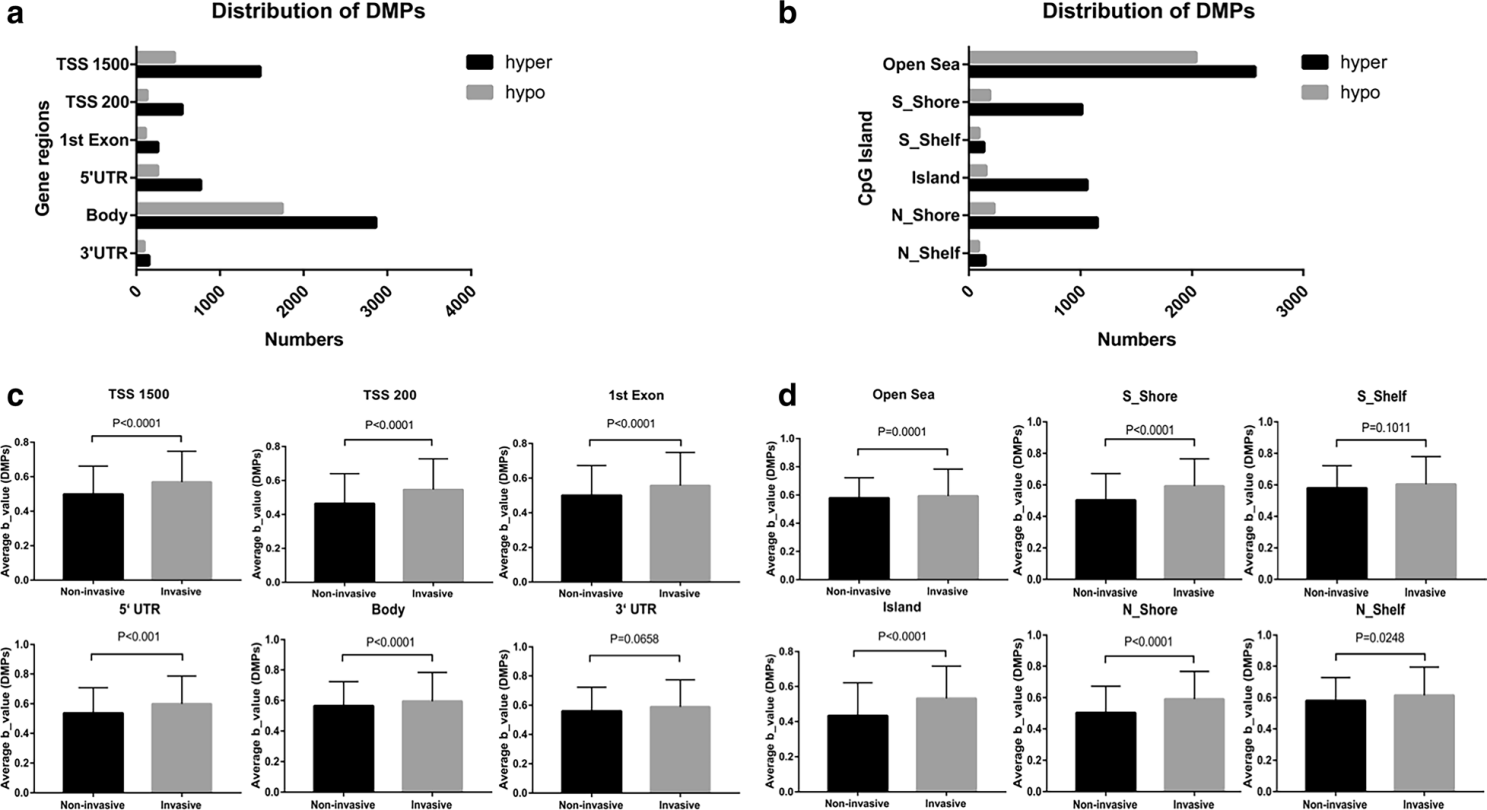
Distributions of differentially methylated probes. **a** Distributions of DMPs based on the genomic region (invasive vs non-invasive tumor). **b** Distributions of DMPs based on the locations of CpG island. **c** DNA methylation level variances were significant in the TSS1500, TSS200, 1st Exon, 5′ UTR, and gene body not in the 3′ UTR region. **d** DNA methylation level variances were significant in the open sea, S_Shore, island, N_Shore and N_Shelf but not S_Shelf

Distributions of DMPs were analyzed based on their genomic regions as well as the locations relative to CpG islands. Among all 8842 DMPs, 22% were found to be located in the TSS1500 (transcription start sites 1500), 7% in the TSS200 (transcription start sites 200), 12% in the 5′ UTR (5′ untranslated region), 4% in 1st exon, 52% in gene body and 3% in the 3′ UTR (Fig. 2a, Additional file 1: Table S10). When considering distribution of DMPs relative to the location of the CpG island, 52% were located in the open sea, 13% in the CpG S_Shore, 2% in the CpG S_Shelf, 14% in the CpG island, 16% in the CpG N_Shore and 3% in the CpG N_Shelf (Fig. 2b, Additional file 1: Table S10).

Some differences between overall DNA methylation levels for invasive and non-invasive tumors were observed when the average β-values were compared according to the gene-related regions and annotations to the CpG islands, respectively. Significant variances in DNA methylation level were found in TSS1500, TSS200, 1st exon, 5′ UTR and gene body between the two groups, though there was no significant difference in 3′ UTR region (Fig. 2c). When focusing on the diversity of DNA methylation level according to CpG island location, significant differences were observed in the open sea, S_Shore, island, N_Shore and N_Shelf but not for the S_Shelf (Fig. 2d).

**Fig. 3:**
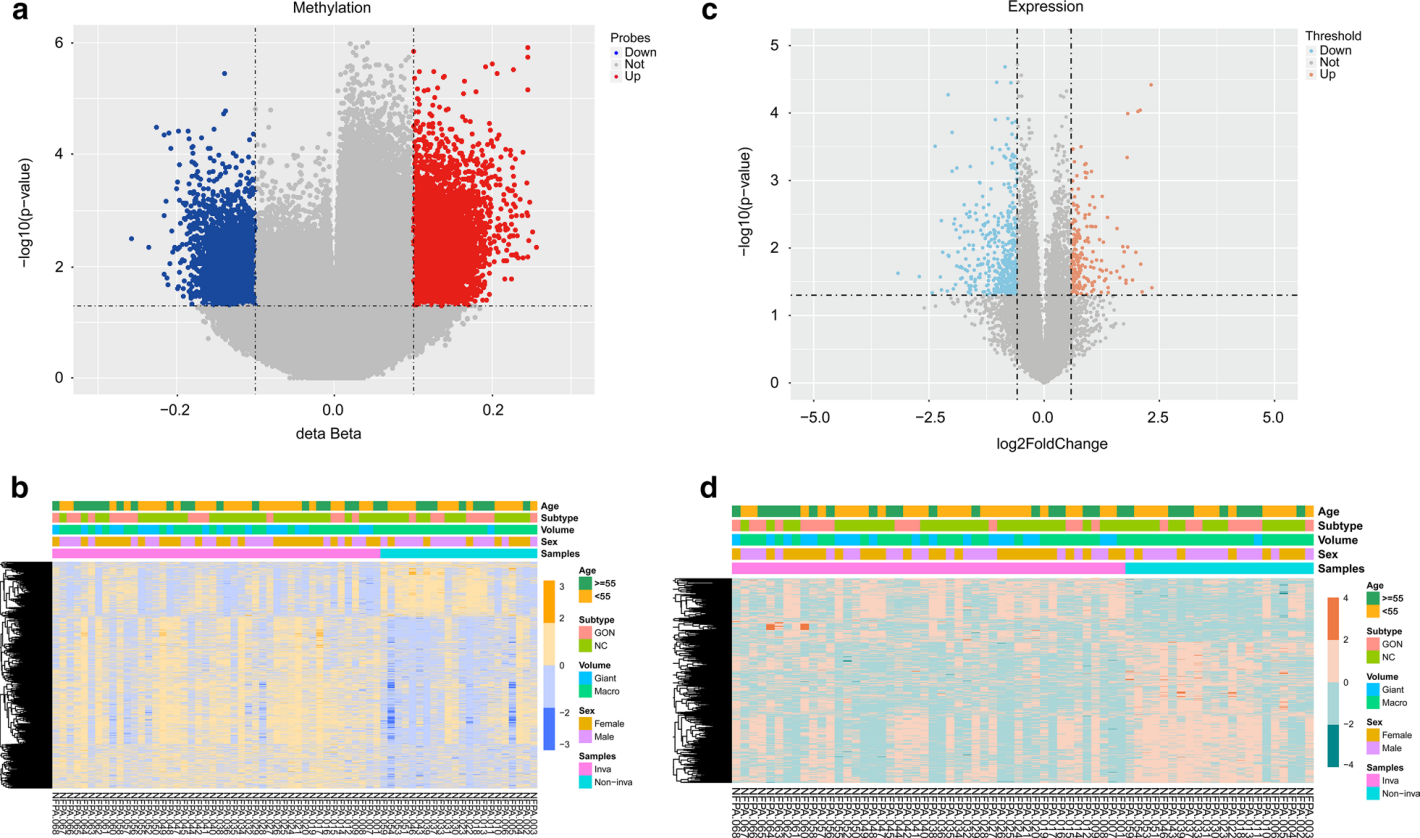
Differential analyses of gene methylation and expression status between invasive and non-invasive tumors. **a** The volcano plot shows 8842 differentially methylated probes (invasive vs non-invasive tumor), indicating 2780 hypomethylated probes (blue) and 6062 hypermethylated probes (red). **b** The heatmap shows the methylation profiles of 68 NFPA samples. The rows represent the different probes and the columns represent each sample. The color in the heatmap represents the methylation level difference, which are hypermethylation (yellow) and hypomethylation (blue). The bar on the top shows the clinical and grouping information, and the patient ID is on the bottom. **c** The volcano plot shows 661 differentially expressed genes, with 206 upregulated genes (red) and 455 downregulated genes (blue). **d** The heatmap shows the expression profiles of the 68 NFPA samples. The rows represent the different genes and the columns represent each sample. The color in the heatmap represents the expression level difference: upregulated (red) and downregulated (green). The bar on the top shows the clinical and grouping information, and the patient ID is on the bottom

Among all 8842 DMPs, there were 6062 hypermethylated and 2780 hypomethylated probes between invasive and non-invasive tumors (Fig. 3a and Additional file 1: Table S2). The heatmap showed the methylation status of all significant probes based on β-values of these CpG sites across all 68 samples (p < 0.05, |∆β| > 0.1, Fig. 3b). We then related the DMPs to specific genes according to their location. A total of 8842 DMPs were related to 4582 genes; for 1904 genes, the DMPs were in the promoter region, and for the remaining 2678 genes, the DMPs were in the non-promoter region (Additional file 1: Table S3).

### Identification of differentially expressed genes

The differential mRNA expression status was also assessed between invasive and non-invasive tumors. A total of 661 genes were differentially expressed (p < 0.05, FDR < 0.25 |log_2_ fold change| > log_2_ 1.5) between two groups, of which, 206 genes were found to be upregulated and 455 genes were downregulated (Fig. 3c and Additional file 1: Table S4). The heatmap and volcano plot of the DEGs are presented in Fig. 3d.

### Integrated analysis of DMGs and DEGs

The DNA methylation changes in the gene promoter region have been demonstrated to be closely associated with gene expression regulation [20, 21]. However, the connection between non-promoter region DNA methylation and gene expression is still in controversial [22–25]. Thus, our ensuing analyses only focused on the DMGs in promoter region with alterations in DNA methylation.

**Fig. 4:**
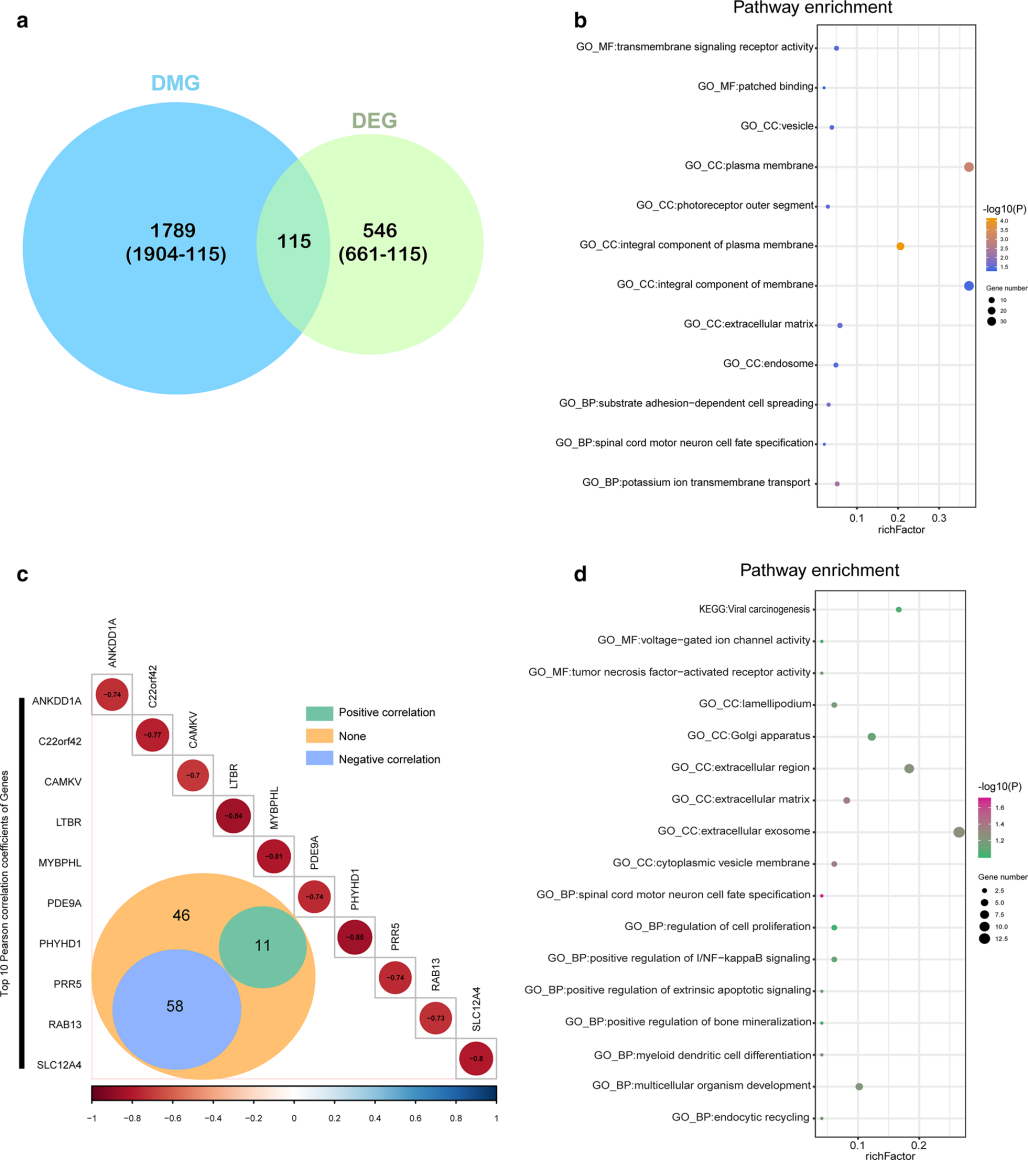
Integrated analysis of DMGs and DEGs. **a** The Venn diagram shows 115 genes with both DNA methylation and expression level changes. **b** GO and KEGG pathway analyses of 115 genes. **c** Pearson analysis of 115 genes. There are 58 genes showing negative correlation (blue), 11 genes showing positive correlation (green) and 46 genes showing no correlation (orange). The R value of the top 10 genes is shown. **d** GO and KEGG pathway analyses of the 58 negative correlation genes

Using the differentially expressed genes based on DMG analyses, we observed 115 of the 661 genes to be both differentially methylated and expressed (Fig. 4a). Enrichment analysis showed that these genes are related to the plasma membrane, integral component of membrane and transmembrane signaling receptor activity etc. (Fig. 4b, Additional file 1: Table S6).

We further studied correlations of the methylation status of the 115 genes and their expression levels using Pearson correlation analysis and found 58 genes with a negative correlation (p < 0.05, R < − 0.2, Fig. 4c, Additional file 1: Table S5). Enrichment analysis of these 58 genes also revealed the relevance of tumor oncogenesis (Fig. 4d, Additional file 1: Table S7).

### Validation of the methylation and expression levels of the candidate genes

Genes with notably negative correlations between DNA methylation and gene expression levels, are involved in various gene ontologies and pathways, or play an important role in different diseases were regarded as candidate genes. Based on the above criteria, we selected 19 genes to validate the invasive behavior of NFPA and performed pyrosequencing and RT-PCR to validate their DNA methylation and expression levels.

**Fig. 5:**
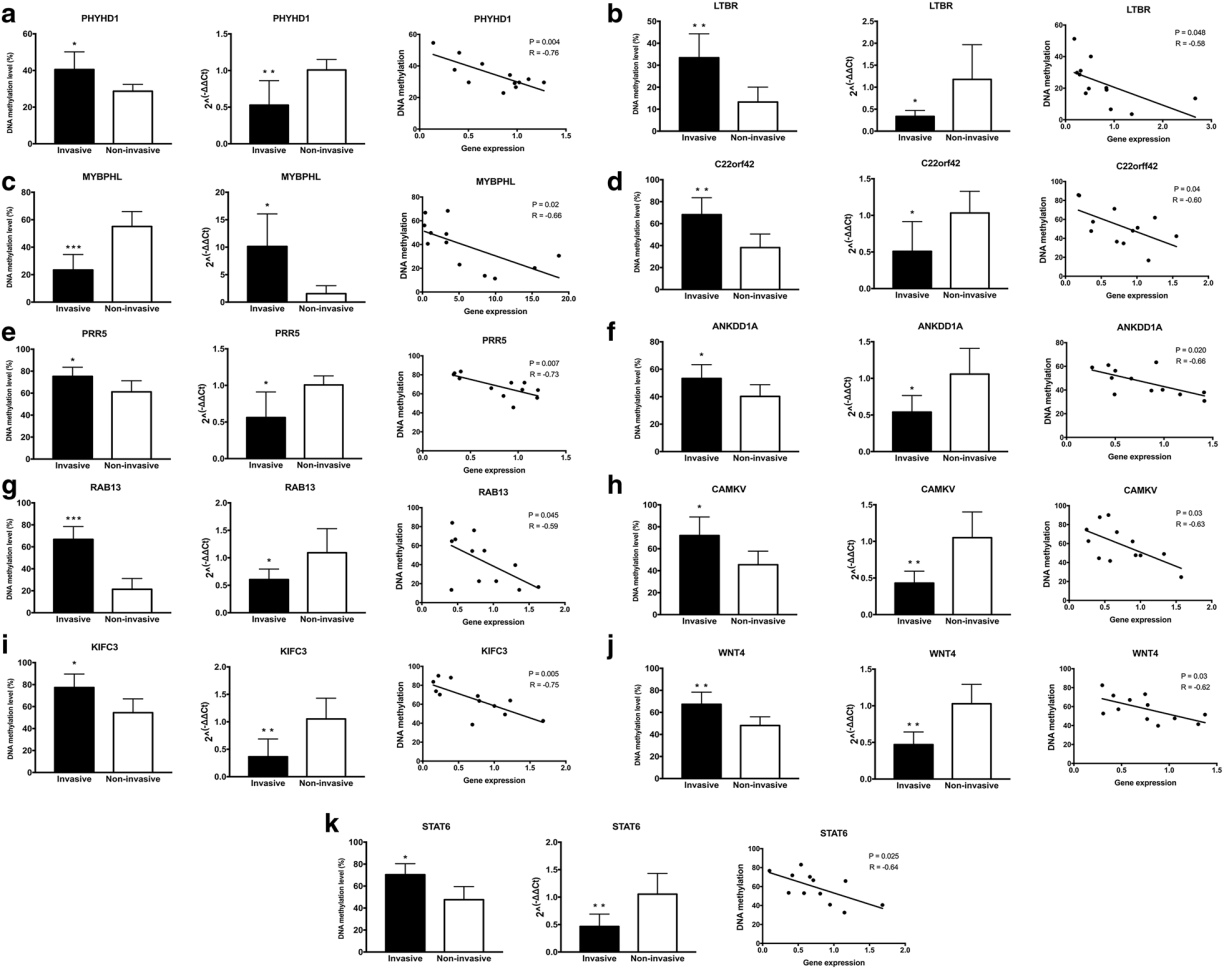
Evaluation of DNA methylation and expression levels of selected genes. The DNA methylation status, expression levels and Pearson correlation of PHYHD1 (**a**), LTBR (**b**), MYBPHL (**c**), C22orf42 (**d**), PRR5 (**e**), ANKDD1A (**f**), RAB13 (**g**), CAMKV (**h**), KIFC3 (**i**), WNT4 (**j**) and STAT6 (**k**) are shown. Each dot represents the average DNA methylation and gene expression level for every sample. *p < 0.05, **p < 0.01, ***p < 0.001

A significant increase in DNA methylation levels in the invasive tumors compared with non-invasive tumors was observed for the following eleven genes: PHYHD1, LTBR, C22orf42, PRR5, ANKDD1A, RAB13, CAMKV, KIFC3, WNT4, STAT6, GBGT1 and GALNT14 (Fig. 5a, b, d–k, Additional file 2: Figure S1e, f). Decreased methylation levels were observed in MYBPHL (Fig. 5c). Conversely, no significant DNA methylation changes were found for SLC12A4, PDE9A, PDLIM4, CHD5, LHX3 and BATF2 (Additional file 2: Figure S1a–d, g, h).

Expression levels of were assessed by RT-PCR using the same tumor samples that were used for pyrosequencing. A significant decrease in the expression level between invasive and non-invasive tumors were observed in following thirteen genes: PHYHD1, LTBR, C22orf42, PRR5, ANKDD1A, RAB13, CAMKV, KIFC3, WNT4, STAT6, SLC12A4, PDE9A and LHX3 (Fig. 5a, b, d–k, Additional file 2: Figure S1a, b, g). In contrast, increased expression w was observed for MYBPHL (Fig. 5c) whereas no significant change in expression was detected for PDLIM4, CHD5, GBGT1, GALNT14 and BATF2 (Additional file 2: Figure S1c–f, h).

Pearson analyses showed a significantly negative correlation between the methylation status and expression levels of PHYHD1, LTBR, MYBPHL, C22orf42, PRR5, ANKDD1A, RAB13, CAMKV, KIFC3, WNT4 and STAT6 (Fig. 5a–k).

These results confirmed that the methylation and expression levels of PHYHD1, LTBR, MYBPHL, C22orf42, PRR5, ANKDD1A, RAB13, CAMKV, KIFC3, WNT4 and STAT6 were consistent with the results of DNA methylation and mRNA microarray analyses. These genes may play a pivotal role in the invasive behavior of NFPA.

## Discussion

Unlike FPA, such as somatotroph adenoma and lactotroph adenoma, there is no targeted medicine for clinical therapy of NFPA, and surgery has been suggested to be the most effective therapeutic approach [5]. Tumor surrounding the internal carotid artery or invading the cavernous sinus are the most frequent factors of a residual tumor, which could be an important risk factor for tumor regrowth [2]. For the above reasons, we focused on alterations in DNA methylation status and corresponding gene expression changes between invasive and non-invasive NFPA, in order to identify key genes involved in invasive behavior and potential candidates for targeted therapy. By integrating whole-genome DNA methylation and gene expression analyses, we identified eleven genes that may correlate with tumor invasion, which may facilitate an understanding of underlying mechanism of pituitary adenoma invasion.

Although germline and somatic mutations are thought to be related to the tumorigenesis of some subtypes of pituitary adenoma, the drivers of NFPA tumorigenesis remain unknown, as are the underlying mechanisms of tumor invasion [26]. PIK3CA mutations have been identified in pituitary adenomas, including lactotroph adenoma, corticotroph adenoma and NFPA. Indeed, such mutations are observed in 8.8% of invasive pituitary tumors, whereas no mutations have been detected in noninvasive tumors [27]. Other studies have found that some genes are closely related to the invasive behavior of pituitary. ESM-1 encodes endocan, which is highly expressed in pituitary adenomas and thought to be related to angiogenesis and strongly associated with tumor invasion in pituitary adenoma [28]. Additionally, Yang et al. [29] found that Caveolin-1 (Cav-1), a principal structural protein of caveolae, promotes pituitary adenoma cell migration and invasion by regulating the interaction between EGR1 and KLF5. Furthermore, ADAM12 overexpression is associated with tumor invasion in pituitary adenoma via the EGFR/ERK signaling pathway [30]. However, to some extent, these studies illustrate the mechanism of tumor invasion from a genetic standpoint, the underlying mechanism has not been fully elucidated.

Previous studies have also illustrated the strong association between pituitary adenoma tumorigenesis or invasion and regulation of DNA methylation. Duong et al. [31] used infinium methylation 27 K arrays to examine DNA methylation in various subtypes of pituitary adenoma and found twelve genes showing methylation alterations, of which three genes, EML2, RHOD and HOXB1, also exhibited significantly decreased expression levels. Ling et al. [32] used genome-scale profiling of DNA methylation in FPA and NFPA and attempted to explore associations with tumor invasion and histopathological subtype. They calculated the mean DNA methylation beta value for the entire filtered probe and did not find significant differences in DNA methylation levels. However, methylation alteration in promoter region is able to affect gene expression and gene body methylation is not always consistent with promoter methylation alteration. As a result, the average beta value of an entire gene may disguise the methylation changes that genuinely affect the gene expression. Although they found that KCNAB2 may contribute to the endocrine-inactive status of NFPA, no DNA methylation changes in were detected between invasive and non-invasive NFPA. Compared with their research, our study focused on the methylation changes in the promoter region between invasive and non-invasive tumor and successfully find eleven genes by integrating analyses of DNA methylation and expression microarray. Some researchers have tried to seek the specific DNA methylation changes in clinically non-functioning pituitary adenoma. For instance, by comparing the DNA methylation profiles between normal pituitary tissue, non-invasive and invasive pituitary adenomas, Kober et al. [33] found that promoter hypermethylation and decreased expression levels of five genes in NFPA compared with normal pituitary. However, differences in the methylation profiles between invasive and non-invasive NFPA were not detected. Additionally, hypermethylation of GALNT9 was found to be significantly downregulated in invasive NFPA, possibly affecting cell adhesion [34].

Differential analysis of all 853,307 probes between invasive and non-invasive tumors enabled us to study the methylation pattern from a more comprehensive perspective. Kober et al. [33] used Illumina HM450K BeadChip to assess the DNA methylation status in NFPA (n = 34) and normal pituitary (n = 4). Meanwhile, they divided 34 NFPA into invasive (n = 18) and non-invasive (n = 16) to explore the DNA methylation alterations. Their research finds methylation and expression differences between NFPA and normal pituitary, which may explain the pathogenesis of NFPA. However, they do not find the methylation differences between invasive and non-invasive NFPA. In our study, we focused on the methylation differences between invasive (n = 46) and non-invasive (n = 20) NFPA and we found hypermethylation appeared to be a more frequent phenomenon in invasive tumors with regard to the distribution of DMPs in genomic regions and the locations of CpG islands. In our study, we used Illumina HM850K BeadChip which contains much more information than HM450K BeadChip, and our chip could find more potential targets. Also, Kober’s research did not perform a whole-genome transcriptome analysis. After they selected target genes from methylation microarray analysis, qRT-RCR was performed to get the expression level of target genes. In our study, we simultaneously perform whole-genome methylation and whole-genome transcriptome analysis which is more comprehensive.

Changes in DNA methylation can be observed in thousands of genes, and only genes with methylation changes that influence expression were considered as candidates. Based on this criterion, we performed whole-genome DNA methylation and gene expression analyses. In this study, we selected key genes showing notably negative correlations between DNA methylation and gene expression levels, are involved in various gene ontologies and pathways, or played an important role in different diseases. We identified PHYHD1, LTBR, MYBPHL, C22orf42, PRR5, ANKDD1A, RAB13, CAMKV, KIFC3, WNT4 and STAT6 as having alterations in DNA methylation and gene expression in invasive NFPA. Most of our genes were reported in large data analysis research, but the significance of methylation changes has not yet been reported. LTBR plays a role in lymphoid development signaling and the immune response, and the hypermethylation of LTBR is observed in odontogenic keratocysts, though its expression is not affected [35]. The relationship between methylation and gene expression is complicated and it can be affected by many factors. In our study, we observed a negative correlation between the hypermethylation of LTBR and its downregulated expression. In addition, hypermethylation of ANKDD1A in glioblastoma has been reported; this gene interacts with FIH1 and decreases HIF1-α stability to inhibit cell autophagy in a hypoxic microenvironment [36, 37], but its function in pituitary adenoma or the significance of promoter methylation is still unclear. RAB13 has been predominantly studied in epithelial cells and its activity regulates tight junction assembly and stimulates cell invasion and migration. Regardless, the methylation regulation of RAB13 has not been reported [38, 39]. A large-scale transcriptomic study found CAMKV to be present in the synaptic neuropil, and quantitative proteomics analysis revealed that CAMKV is downregulated at the synapse upon sensory deprivation, though changes in methylation in tumors have not been explored yet [40, 41]. The overexpression of WNT4 has been observed in various subtypes of pituitary adenoma, but it displays an inverse correlation with tumor invasion [42]. In the present study, decreased expression of WNT4 was observed and our results showed that DNA methylation may play a role in its regulation. In general, alterations in methylation of most of the genes identified in our study have not been examined in pituitary adenoma or other solid tumors, and further research on their function and mechanism in tumor invasion is essential.

Promoter methylation alterations have been proved to be closely related to gene expression. In pituitary adenoma, the methylation alteration of LAMA2, GALNT9 and DAP methylation has been proven to be related to tumor invasion [34, 43, 44]. In our study, alteration in promoter DNA methylation of eleven genes was associated with NFPA invasion. However, recent studies have reported that the methylation changes in gene body regions may also result in the gene expression changes. Yang et al. [25] found that gene body DNA methylation increases gene expression and this regulation is dependent on the presence of the DNMT3B. Moreover, hypermethylation in the gene body region accompanied by upregulated expression was also observed in a hepatocellular carcinoma mouse model [22], and Chen et al. [23] found gene body hypermethylation to be significantly associated with silencing of the tumor-related genes in kidney cancer. Though whole-genome bisulfite sequencing analysis of multiple individuals, Lou et al. [24] revealed that gene body hypermethylation leads to reduced transcription efficiency. Regardless, the correlation between gene body methylation and gene expression is still controversial. Therefore, we selected genes that showed a negative correlation between promoter methylation and gene expression and did not further investigate differential probes located in gene body regions. This may be one limitation of our study, and our future research may focus on the regulation mechanism between gene body methylation and expression in NFPA.

## Conclusions

In conclusion, this study integrates DNA methylation and gene expression and successfully identified key genes in invasive NFPA. We found alterations of DNA methylation in the promoter region and expression changes for eleven genes between invasive and non-invasive NFPA. Our study may promote the understanding of NFPA invasion and facilitate targeted therapy for patients with invasive tumors.

## Supplementary information

**Additional file 1: Table S1.** Clinical information of 68 patients with NFPA. **Table S2.** List of differentially methylated probes. **Table S3.** List of differentially methylated genes in the promoter region. **Table S4.** List of differentially expressed genes. **Table S5.** Pearson analyses results of 69 significant genes. **Table S6.** GO and KEGG analyses of 115 genes. **Table S7.** GO and KEGG analyses of 58 genes. **Table S8.** Pyrosequencing primers of 19 genes. **Table S9.** RT-PCR primers of 20 genes (including thr control). **Table S10.** Distribution of differential methylated probes.

**Additional file 2: Figure S1.** The DNA methylation status and expression levels of SLC12A4, PDE9A, PDLIM4, CHD5, GNGT1, GALNT14, LHX3 and BATF2. *p < 0.05, **p < 0.01.

## Data Availability

The datasets during and/or analyzed during the current study are available from the corresponding author on a reasonable request.

## Abbreviations

PA: pituitary adenoma
NFPA: clinically non-functioning pituitary adenoma
PCC: Pearson correlation co-efficient
DMPs: differentially methylated probes
DMGs: differentially methylated genes
DEGs: differentially expressed genes

## References

[1] Alexander JM, Biller BM, Bikkal H, Zervas NT, Arnold A, Klibanski A (1990). Clinically nonfunctioning pituitary tumors are monoclonal in origin. J Clin Invest.

[2] Ostrom QT, Gittleman H, Truitt G, Boscia A, Kruchko C, Barnholtz-Sloan JS (2018). CBTRUS statistical report: primary brain and other central nervous system tumors diagnosed in the United States in 2011–2015. Neuro Oncol.

[3] Molitch ME (2012). Management of incidentally found nonfunctional pituitary tumors. Neurosurg Clin N Am.

[4] Meij BP, Lopes MB, Ellegala DB, Alden TD, Laws ER (2002). The long-term significance of microscopic dural invasion in 354 patients with pituitary adenomas treated with transsphenoidal surgery. J Neurosurg.

[5] Melmed S (2008). Update in pituitary disease. J Clin Endocrinol Metab.

[6] Newey PJ, Nesbit MA, Rimmer AJ, Head RA, Gorvin CM, Attar M, Gregory L, Wass JA, Buck D, Karavitaki N (2013). Whole-exome sequencing studies of nonfunctioning pituitary adenomas. J Clin Endocrinol Metab.

[7] Sonabend AM, Musleh W, Lesniak MS (2006). Oncogenesis and mutagenesis of pituitary tumors. Expert Rev Anticancer Ther.

[8] Dawson MA, Kouzarides T (2012). Cancer epigenetics: from mechanism to therapy. Cell.

[9] De Jager PL, Srivastava G, Lunnon K, Burgess J, Schalkwyk LC, Yu L, Eaton ML, Keenan BT, Ernst J, McCabe C (2014). Alzheimer’s disease: early alterations in brain DNA methylation at ANK1, BIN1, RHBDF2 and other loci. Nat Neurosci.

[10] Fasanelli F, Baglietto L, Ponzi E, Guida F, Campanella G, Johansson M, Grankvist K, Johansson M, Assumma MB, Naccarati A (2015). Hypomethylation of smoking-related genes is associated with future lung cancer in four prospective cohorts. Nat Commun.

[11] Pellacani D, Droop AP, Frame FM, Simms MS, Mann VM, Collins AT, Eaves CJ, Maitland NJ (2018). Phenotype-independent DNA methylation changes in prostate cancer. Br J Cancer.

[12] Simpson DJ, Hibberts NA, McNicol AM, Clayton RN, Farrell WE (2000). Loss of pRb expression in pituitary adenomas is associated with methylation of the RB1 CpG island. Cancer Res.

[13] Simpson DJ, Bicknell JE, McNicol AM, Clayton RN, Farrell WE (1999). Hypermethylation of the p16/CDKN2A/MTSI gene and loss of protein expression is associated with nonfunctional pituitary adenomas but not somatotrophinomas. Genes Chromosomes Cancer.

[14] Knosp E, Steiner E, Kitz K, Matula C (1993). Pituitary adenomas with invasion of the cavernous sinus space: a magnetic resonance imaging classification compared with surgical findings. Neurosurgery.

[15] Nordlund J, Backlin CL, Wahlberg P, Busche S, Berglund EC, Eloranta ML, Flaegstad T, Forestier E, Frost BM, Harila-Saari A (2013). Genome-wide signatures of differential DNA methylation in pediatric acute lymphoblastic leukemia. Genome Biol.

[16] Aryee MJ, Jaffe AE, Corrada-Bravo H, Ladd-Acosta C, Feinberg AP, Hansen KD, Irizarry RA (2014). Minfi: a flexible and comprehensive Bioconductor package for the analysis of Infinium DNA methylation microarrays. Bioinformatics.

[17] Ritchie ME, Phipson B, Wu D, Hu Y, Law CW, Shi W, Smyth GK (2015). limma powers differential expression analyses for RNA-sequencing and microarray studies. Nucleic Acids Res.

[18] Li CQ, Huang GW, Wu ZY, Xu YJ, Li XC, Xue YJ, Zhu Y, Zhao JM, Li M, Zhang J (2017). Integrative analyses of transcriptome sequencing identify novel functional lncRNAs in esophageal squamous cell carcinoma. Oncogenesis.

[19] Livak KJ, Schmittgen TD (2001). Analysis of relative gene expression data using real-time quantitative PCR and the 2(-Delta Delta C(T)) Method. Methods.

[20] Kucuk C, Hu X, Jiang B, Klinkebiel D, Geng H, Gong Q, Bouska A, Iqbal J, Gaulard P, McKeithan TW, Chan WC (2015). Global promoter methylation analysis reveals novel candidate tumor suppressor genes in natural killer cell lymphoma. Clin Cancer Res.

[21] Kulis M, Esteller M (2010). DNA methylation and cancer. Adv Genet.

[22] Arechederra M, Daian F, Yim A, Bazai SK, Richelme S, Dono R, Saurin AJ, Habermann BH, Maina F (2018). Hypermethylation of gene body CpG islands predicts high dosage of functional oncogenes in liver cancer. Nat Commun.

[23] Chen K, Zhang J, Guo Z, Ma Q, Xu Z, Zhou Y, Xu Z, Li Z, Liu Y, Ye X (2016). Loss of 5-hydroxymethylcytosine is linked to gene body hypermethylation in kidney cancer. Cell Res.

[24] Lou S, Lee HM, Qin H, Li JW, Gao Z, Liu X, Chan LL, Kl Lam V, So WY, Wang Y (2014). Whole-genome bisulfite sequencing of multiple individuals reveals complementary roles of promoter and gene body methylation in transcriptional regulation. Genome Biol.

[25] Yang X, Han H, De Carvalho DD, Lay FD, Jones PA, Liang G (2014). Gene body methylation can alter gene expression and is a therapeutic target in cancer. Cancer Cell.

[26] Fukuoka H, Takahashi Y (2014). The role of genetic and epigenetic changes in pituitary tumorigenesis. Neurol Med Chir.

[27] Lin Y, Jiang X, Shen Y, Li M, Ma H, Xing M, Lu Y (2009). Frequent mutations and amplifications of the PIK3CA gene in pituitary tumors. Endocr Relat Cancer.

[28] Cornelius A, Cortet-Rudelli C, Assaker R, Kerdraon O, Gevaert MH, Prevot V, Lassalle P, Trouillas J, Delehedde M, Maurage CA (2012). Endothelial expression of endocan is strongly associated with tumor progression in pituitary adenoma. Brain Pathol.

[29] Yang W, Xu T, Qiu P, Xu G (2018). Caveolin-1 promotes pituitary adenoma cells migration and invasion by regulating the interaction between EGR1 and KLF5. Exp Cell Res.

[30] Wang J, Zhang Z, Li R, Mao F, Sun W, Chen J, Zhang H, Bartsch JW, Shu K, Lei T (2018). ADAM12 induces EMT and promotes cell migration, invasion and proliferation in pituitary adenomas via EGFR/ERK signaling pathway. Biomed Pharmacother.

[31] Duong CV, Emes RD, Wessely F, Yacqub-Usman K, Clayton RN, Farrell WE (2012). Quantitative, genome-wide analysis of the DNA methylome in sporadic pituitary adenomas. Endocr Relat Cancer.

[32] Ling C, Pease M, Shi L, Punj V, Shiroishi MS, Commins D, Weisenberger DJ, Wang K, Zada G (2014). A pilot genome-scale profiling of DNA methylation in sporadic pituitary macroadenomas: association with tumor invasion and histopathological subtype. PLoS ONE.

[33] Kober P, Boresowicz J, Rusetska N, Maksymowicz M, Goryca K, Kunicki J, Bonicki W, Siedlecki JA, Bujko M (2018). DNA methylation profiling in nonfunctioning pituitary adenomas. Mol Cell Endocrinol.

[34] Gu Y, Zhou X, Hu F, Yu Y, Xie T, Huang Y, Zhao X, Zhang X (2016). Differential DNA methylome profiling of nonfunctioning pituitary adenomas suggesting tumour invasion is correlated with cell adhesion. J Neurooncol.

[35] Pereira KMA, Costa S, Pereira NB, Diniz MG, Castro WH, Gomes CC, Gomez RS (2017). DNA methylation profiles of 22 apoptosis-related genes in odontogenic keratocysts before and after marsupialization. Oral Surg Oral Med Oral Pathol Oral Radiol.

[36] Feng J, Zhang Y, She X, Sun Y, Fan L, Ren X, Fu H, Liu C, Li P, Zhao C (2019). Hypermethylated gene ANKDD1A is a candidate tumor suppressor that interacts with FIH1 and decreases HIF1alpha stability to inhibit cell autophagy in the glioblastoma multiforme hypoxia microenvironment. Oncogene.

[37] Zhang Z, Tang H, Wang Z, Zhang B, Liu W, Lu H, Xiao L, Liu X, Wang R, Li X (2011). MiR-185 targets the DNA methyltransferases 1 and regulates global DNA methylation in human glioma. Mol Cancer.

[38] Ioannou MS, Bell ES, Girard M, Chaineau M, Hamlin JN, Daubaras M, Monast A, Park M, Hodgson L, McPherson PS (2015). DENND2B activates Rab13 at the leading edge of migrating cells and promotes metastatic behavior. J Cell Biol.

[39] Marzesco AM, Dunia I, Pandjaitan R, Recouvreur M, Dauzonne D, Benedetti EL, Louvard D, Zahraoui A (2002). The small GTPase Rab13 regulates assembly of functional tight junctions in epithelial cells. Mol Biol Cell.

[40] Butko MT, Savas JN, Friedman B, Delahunty C, Ebner F, Yates JR, Tsien RY (2013). In vivo quantitative proteomics of somatosensory cortical synapses shows which protein levels are modulated by sensory deprivation. Proc Natl Acad Sci USA.

[41] Cajigas IJ, Tushev G, Will TJ, S tom Dieck, Fuerst N, Schuman EM (2012). The local transcriptome in the synaptic neuropil revealed by deep sequencing and high-resolution imaging. Neuron.

[42] Li W, Zhang Y, Zhang M, Huang G, Zhang Q (2014). Wnt4 is overexpressed in human pituitary adenomas and is associated with tumor invasion. J Clin Neurosci.

[43] Simpson DJ, Clayton RN, Farrell WE (2002). Preferential loss of death associated protein kinase expression in invasive pituitary tumours is associated with either CpG island methylation or homozygous deletion. Oncogene.

[44] Wang RQ, Lan YL, Lou JC, Lyu YZ, Hao YC, Su QF, Ma BB, Yuan ZB, Yu ZK, Zhang HQ (2019). Expression and methylation status of LAMA2 are associated with the invasiveness of nonfunctioning PitNET. Ther Adv Endocrinol Metab.

