# Supplementary figures and images for: Identification of key genes in invasive clinically non-functioning pituitary adenoma by integrating analysis of DNA methylation and mRNA expression profiles
Source: J Transl Med. 2019 Dec 3;17:407. doi: 10.1186/s12967-019-02148-3 (PMC6892283; doi:10.1186/s12967-019-02148-3)

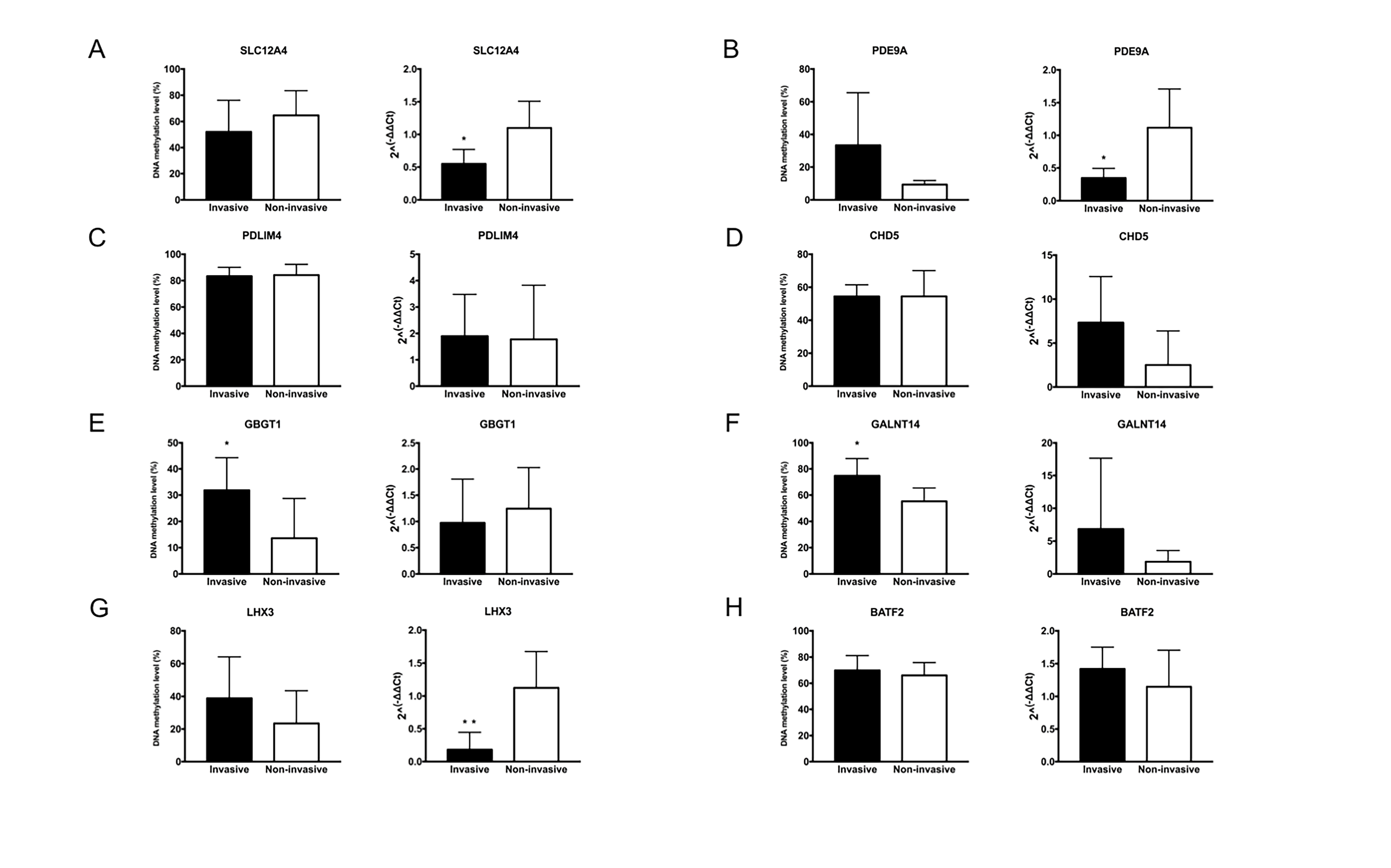

Supplement: Supplementary file 2 — Additional file 2: Figure S1. The DNA methylation status and expression levels of SLC12A4, PDE9A, PDLIM4, CHD5, GNGT1, GALNT14, LHX3 and BATF2. *p < 0.05, **p < 0.01. [file 12967_2019_2148_MOESM2_ESM.tif]
